# Pneumoperitoneum in a non-acute abdomen—pneumatosis cystoides intestinalis

**DOI:** 10.1186/s40792-015-0046-0

**Published:** 2015-05-28

**Authors:** Siang Mei Sally Ooi

**Affiliations:** Liverpool Hospital, Elizabeth Street, Liverpool, NSW 2170 Australia

**Keywords:** Pneumatosis cystoides intestinalis, Pneumoperitoneum, Bowel perforation, Bowel dilatation, Intestinal cysts, Extra-luminal gas

## Abstract

**Aim:**

The aim of this study is to increase the understanding of pneumatosis cystoides intestinalis (PCI) and its incidents.

**Method:**

We report here a case of PCI in an 88-year-old man with a provisional diagnosis of perforated viscus and possible ischaemic bowels based on CT findings of pneumoperitoneum. The patient was found to have extensive PCI on his small bowels. We then systematically search the PubMed database for case reports for articles containing ‘pneumatosis intestinalis’ in their titles or key words.

**Results:**

The study group consisted of 52 cases on PCI from the period of 2010–2014 with the focus on the adult population. The youngest patient was 18 years old and the oldest was 91 years old. The mean age was 60.4 years (range, 18–91 years old). There were 27 (52 %) females and 25 (48 %) males. The most common symptoms were abdominal pain (79 %) followed by nausea/vomiting (27 %) and abdominal distension (19 %). CT imaging was the most common investigation modality used (94 %). Three (6 %) of the patients had laparoscopic treatment while 20 (38 %) had laparotomy. Thirty-six (69 %) of them recovered uneventfully while 9 (17 %) of the patients died.

**Conclusion:**

Although there have been more case reports published on PCI in the recent years, the understanding of this condition remains in the infancy stage. PCI can be difficult to diagnose and can be easily misdiagnosed as pneumoperitoneum in an acute abdomen. Often it is identified incidentally during operation. Asymptomatic PCI should be treated conservatively, while emergency laparotomy should be reserved for life threatening abdominal pathology.

## Background

Pneumatosis cystoides intestinalis (PCI) is an uncommon condition where intramural gas cysts are distributed in the small bowel, although the large bowel may also be involved. There is no clear etiology with several proposed theories including mechanical, pulmonary, and bacterial. PCI does not usually cause symptoms and is often incidental on radiology imaging or intra-operative finding. We present a case of an 88-year-old man who was transferred from a regional hospital to a tertiary hospital with a probable diagnosis of perforated hollow viscus.

## Case presentation

**Fig 1:**
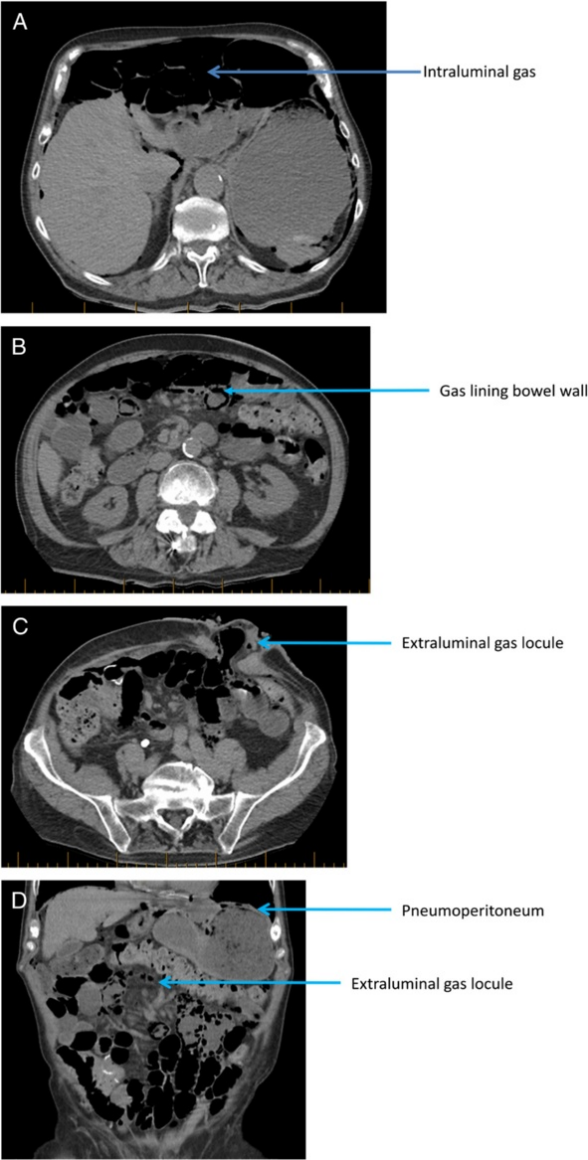
CT scan shows diffuse intramural gas with suspected pneumoperitoneum. **a** Collection of intaluminal gas in PCI appearing like pneumoperitoneum. **b** Gas lining the bowel wall. **c** PCI appearing like extraluminal gas locules. **d** PCI appering like extraluminal gas locules and pneumoperitoneum

An 88-year-old man presented to a regional hospital with 3 days of generalised abdominal pain with nausea, vomiting, and obstipation. His past medical history included prostate carcinoma treated with radiotherapy, Hartman’s procedure, and multiple episodes of small-bowel obstruction requiring bowel resection, atrial fibrillation, aortic stenosis, hypertension, and cerebrovascular disease. A computerised tomography (CT) scan performed at the regional hospital showed diffuse intramural gas with suspected pneumoperitoneum (Fig. 1a–d). The patient was immediately transferred to a tertiary hospital for further management.

Upon arrival at the tertiary hospital, he was haemodynamically stable. The patient had mild generalised abdominal pain, with a soft distended abdomen. There were no signs of peritonism. The stoma was healthy looking but had no output. The patient was noted to have a nasogastric tube with minimal output and an indwelling catheter with adequate urine output. Based on CT images of pneumoperitoneum, he was taken to the theatre with a provisional diagnosis of perforated viscus and possible ischaemic bowels.

**Fig. 2:**
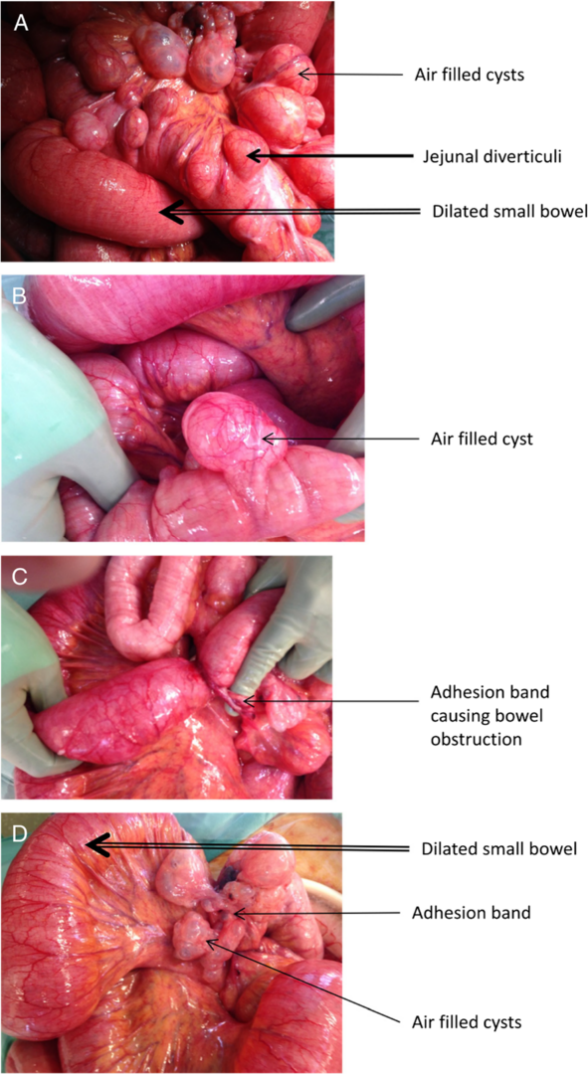
**a**–**b** Multiple air-filled cysts consistent with appearance of pneumatosis cystoids intestinalis (*thin arrow*) and jejuna diverticuli (*thick arrow*). **a** also shows dilated small bowel loops of up to 7 cm (*double line arrow*). **c**–**d** Bands of adhesions, some causing small bowel obstruction

At laparotomy, there was 1.5 L of serous peritoneal fluid with no faecal contamination. There were multiple adhesion bands causing small bowel obstruction with transition point in distal ileal segment; numerous segments of air-filled cysts and large jejunal diverticuli were identified on an incidental finding. Volvulus caused by the adhesion bands was released (Fig. 2a–d). The bowel was mechanically decompressed and 2 L of faeculant material was drained via nasogastric tube. No clinical signs of communication between the cysts and bowel lumen were demonstrated. There was no perforation and no bowel ischaemia.

**Fig. 3:**
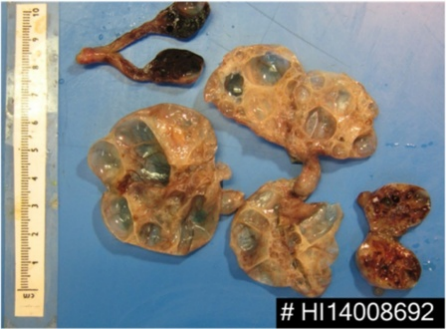
Resected pneumatosis cysts cut open with multilobulated appearance

Seven of the cystic lesions from the small bowel were excised for histological analysis. Histology was consistent with PCI. Macroscopically, the outer surface of the cystic masses was multi-lobulated, thin, and shiny. The outer surface also showed dilated blood vessels; sectioning of the cystic masses revealed multiple gas filled cystic spaces 1 to 20 mm in diameter (Fig. 3).

**Fig. 4:**
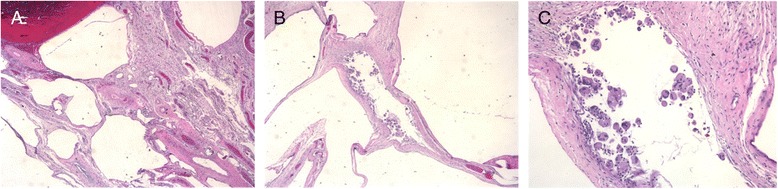
Low power microscopy showing variable-sized cystic spaces. **a** Low power microscopy showing variable sized cystic spaces. **b** Low power microscopy showing variable sized cystic spaces. **c** High power microscopy showing histiocytes and foreign body type giant cells lining the cystic spaces

Microscopically, the specimens have variable-sized cystic spaces lined by histiocytes and multinucleated foreign body giant cells (Fig. 4a–c), on a background of dilated and congested blood vessels. Areas of fresh and old haemorrhage with hemosiderin laden macrophages were noted. There was no evidence of malignancy.

### Search strategy

A search was performed through PubMed database in the last 5 years (2010–2014) on case reports containing ‘pneumatosis intestinalis’ in the titles or key words. Only articles written in English and available in full were included in our analysis.

## Results

The study group included 52 case reports relating to the adult populations. There were 27 (52 %) females and 25 (48 %) males. The mean age was 60.4 years (range, 18–91 years old). Thirteen patients (25 %) fell within the age group of 60–69 years old. Nine patients (17 %) were between 70–79 years old and another 9 patients (17 %) were between 80–89 years old. The most common symptom was abdominal pain (*n* = 41, 79 %) followed by nausea and vomiting (*n* = 14, 27 %), abdominal distension (*n* = 10, 19 %), per rectal bleeding or bloody stools (*n* = 7, 13 %), diarrhoea (*n* = 6, 12 %), and constipation (*n* = 6, 12 %). During examinations, 6 patients had a soft abdomen with no tenderness while 15 patients were found to have peritonitic signs. CT investigations were performed on 49 (94 %) patients while abdominal X-rays were performed on 17 (33 %). Colonoscopy was performed on 10 patients as a form of investigation. Twenty-seven (52 %) patients were treated conservatively with medical therapy, such as antibiotics, hyperbaric oxygen therapy, bowel rest, and observation. Three (6 %) patients underwent laparoscopy, while 20 (38 %) received laparotomy. Only 1 patient who had laparoscopy needed to be converted to laparotomy. In terms of outcome, 36 (69 %) recovered uneventfully. Nine (17 %) of them died, of whom 2 died prior to surgery, and the remaining 7 died postoperatively. Amongst the 9 deceased patients, 4 died from multi-organ failures, 2 died from sepsis and septic shock, 2 died from pulmonary complications, and 1 died from hypovolemic shock after surgery with autopsy showing intra mural haemorrhage. Both the patients who died from sepsis and septic shock were deceased before surgery.

## Discussion

PCI, also known as intramural gas, refers to gas within the bowel wall. Other names include pseudopneumatosis, intestinal emphysema, and bullous emphysema of intestines [1]. In our discussion, PCI and intramural air will be used interchangeably. The condition was first documented by Du Vernoi in 1783 [1, 2]. It is not a common finding with little research on this topic, although in recent years there have been more case reports.

### Causes

Based on current studies, there is no definite etiology of PCI. PCI appears to be associated with mechanical, bacterial, and biochemical processes. Some papers suggest an association of PCI with immunosuppressed patients [1, 3].

The mechanical theory proposes that increased intra-luminal pressure forces gas within the bowel lumen to breach the mucosal or serosal layers [1, 4, 5]. Once inside the mucosal layer, the gas travels along the mesenteric blood vessels. The gas may continue along the mesentery to distal sites along the entire bowel, which then accumulates to form cysts. Often, gas in the bowel lumen is produced by gastrointestinal pathologies as a result of ischaemia or inflammation. In a study of 50 patients with Crohn’s disease, 6 patients were found to have pneumatosis intestinalis while there were none in the control group (i.e. patients without Crohn’s disease) [3]. However, the mechanical theory cannot explain how cysts persist after formation [4].

The pulmonary theory refers to increased intra-luminal pressure due to the respiratory system [2, 4, 6]. In patients with chronic obstructive pulmonary disease, constant coughing causes the alveoli to rupture. Gas from the alveoli dissects along the aorta within the mediastinum and through the diaphragm to the mesenteric blood vessels. Gas in the mesenteric blood vessels breaches the bowel wall and subsequently gets trapped in the bowel wall forming cysts. However, this theory fails to explain the presence of high levels of hydrogen within the intramural cysts [4].

In the bacterial theory, gas is introduced into the bowel lumen by pathogens. The gas again breaches the bowel wall integrity and seeps into the mucosal or serosal layer [1, 4, 5]. However, studies have shown that the gas in the cysts is sterile. If the cysts were to rupture, it causes pneumoperitoneum, and the extra-luminal gas is not known to cause peritonism. Hence, the bacterial theory may not explain the etiology of PCI.

Finally, the biochemical theory describes the production of hydrogen gas during metabolism of food groups, especially carbohydrate. The increased amount of gaseous by-product raises intra-luminal pressure and the gas is forced into the weakened bowel wall. Trapped extra-luminal gas accumulates to form cysts.

Several case reports have postulated a relationship of PCI with patients who are immunosuppressed [7]. PCI has been found in patients on corticosteroids such as those with Crohn’s disease and ulcerative colitis [1–3, 8]. Others have found a relationship between PCI and those who have undergone chemotherapy [5]. No explanation is available so far on how corticosteroids predispose to PCI.

Many hypotheses have been discussed in the literatures, while none can fully explain the etiology of PCI. At present, the condition is linked to several pathologies as described above. However, it must be stressed that PCI is not a disease but a physiological reaction to several conditions; hence, there is no single etiology [4, 9].

### Clinical features

PCI is usually a benign, asymptomatic, and occult condition. Review of the existing literature shows that patients who are symptomatic present in a diverse manner as a result of the underlying cause of PCI or complications from PCI. Due to the vague symptoms, the condition is easily misdiagnosed as bowel ischaemia or infarction [10]. In this case, what appeared to be pneumoperitoneum has been misdiagnosed as a perforated viscus. He was found to have small bowel obstruction caused by adhesion bands, and PCI was discovered incidentally during operation. Symptoms vary enormously amongst patients and are often vague [2, 6]. The majority of patients have abdominal pain, abdominal distension, nausea and vomiting, and a change in bowel habit [1, 4]. A small number of patients complain of excessive flatulence, tenemus, loss of appetite, and weight loss. These non-specific symptoms can easily lead to a misdiagnosis of irritable bowel syndrome. Physical examination can be completely normal or reveal mild generalised abdominal distension and tenderness. PCI can lead to complications like small or large bowel obstruction, volvulus, intussusception, and intra-abdominal haemorrhage. Generally, this benign condition is diagnosed incidentally during operation.

### Investigations

As illustrated in our case, PCI can easily mimic pneumoperitoneum on radiological imaging. Intramural air may appear as radiolucent shadows along the bowel lumen on X-ray [9]. Extensive PCI or ruptured cysts may appear like free air under the diaphragm in an erect chest X-ray. CT being the more sensitive imaging may show circumferential gas collections outside the bowel lumen [1] or reveal life-threatening events related to PCI like bowel ischaemia [11]. Two features of PCI have been described in the literature—cystic circumferential gas is associated with good prognosis, and linear gas is associated with poor prognosis [2, 11]. CT can also identify co-existing conditions like colitis, perforation, obstruction, and neoplasms. Although CT is deemed to be the most sensitive imaging modality, PCI can give the same appearance as intra-faecal gas, gas in pseudopolyps, and extra-luminal gas [3]. Ultrasound shows high amplitude gas echoes with acoustic shadowing. Barium study shows protrusion of submucosa causing filling defects in the intestinal lumen. Endoscopically, PCI appears as submucosal cysts with pale or bluish tint. When biopsied, PCI may rapidly deflate with an audible hiss [8].

Due to diverse interpretation, imaging should not be used for definitive diagnosis. Up to 27 % of benign PCI was misdiagnosed as surgical abdomen resulting in unnecessary operation [10]. Intra-operative finding of PCI is the most sensitive method of diagnosis.

### Histology findings

#### Typical macroscopic appearance

Macroscopically, PCI is characterised by submucosal cysts varying from a few millimetres to centimetres in diameter [8, 10]. They may appear as a singular cyst or a branching pattern. Furthermore, they protrude into the bowel lumen. The cystic lesions have an outer layer with a bluish tint. Subserosal cysts are generally formed adjacent to the mesenteric blood vessels.

#### Typical microscopic appearance

PCI are true pseudocysts with no epithelial lining. Histiocytes and multinucleated giant cells line the submucosal or subserosal spaces and rarely the muscularis layer [8]. These cells are commonly found in inflammatory conditions such as Crohn’s disease and colitis. The distinguishing feature for PCI is that the giant cells usually line a rounded or cleft-like space. Unlike inflammatory processes, a pathogen is usually not found in PCI. Another distinctive feature is the presence of round empty space in the submucosa resembling fat. Non-specific findings are inflammation, eosinophilia, gland disarray, vascular ectasia, and edema [10, 12].

### Management

An incidental finding of PCI is usually benign and does not require treatment. The important decision is recognising when to perform an emergency laparotomy and when to manage conservatively. Patients who do not require surgery can be managed with medical therapies like high flow oxygen, hyperbaric therapy, antibiotics and special elemental diets [1]. Surgery is generally reserved for management of primary etiology, such as bowel obstruction and bowel ischaemia. Surgery may be elected in cases where medical therapies have failed. Laparotomy should be avoided in primary PCI as an unnecessary operation can lead to a fatal outcome [2, 10]. Uncomplicated PCI can be safely managed conservatively [4].

### Further studies

PCI is an uncommon and poorly understood condition despite being first described in 1738. Identifying the underlying pathophysiology of PCI could potentially avoid misdiagnosis and mismanagement. The majority of the published literatures are case reports. To date, there has been no randomised controlled trial and very few literature reviews. Wu and his colleagues published a systemic analysis in 2013. They also found no studies on epidemiology or randomised control trials on PCI. They performed a systematic review of 239 PCI cases and found that it is a condition more common in males than females and more frequent in large bowel than small bowel [4]. Further studies are justified for every aspect of PCI from epidemiology, etiology, presentations, diagnosis, and treatments to prognosis.

## Conclusions

Pneumatosis cystoides intestinalis refers to intramural gas which often presents as an incidental finding in benign cases. The exact cause of PCI is unknown, but the mechanical theory appears to be the most popular. It has been associated with gastrointestinal and respiratory complications. CT imaging appears to be the most sensitive radiological modality for diagnosis. The condition can easily be misdiagnosed as an acute abdomen leading to unnecessary surgery. From a clinical perspective, it is essential not to confuse the incidental finding of asymptomatic pneumatosis with symptomatic colonic perforation because the treatment is significantly different. As illustrated in our case, clinical presentations are very vague, mimicking several other intra-abdominal pathologies. Benign PCI should always be treated conservatively. Emergency laparotomy is reserved for life-threatening abdominal pathology. The typical microscopic appearance of PCI shows multinucleated giant cells lining the air-filled space. Although PCI was first documented more than 200 years ago, it is still an under researched topic and further studies and literature reviews are justified.

## Consent

Written informed consent was obtained from the patient for publication of this case report and any accompanying images. A copy of the written consent is available for review by the Editor-in-Chief of this journal.
